# In-silico guided chemical exploration of KDM4A fragments hits

**DOI:** 10.1186/s13148-023-01613-7

**Published:** 2023-12-21

**Authors:** Jessica Lombino, Rosario Vallone, Maura Cimino, Maria Rita Gulotta, Giada De Simone, Maria Agnese Morando, Raffaele Sabbatella, Simona Di Martino, Mario Fogazza, Federica Sarno, Claudia Coronnello, Maria De Rosa, Chiara Cipollina, Lucia Altucci, Ugo Perricone, Caterina Alfano

**Affiliations:** 1 Molecular Informatics Group, Fondazione Ri.MED, 90100 Palermo, Italy; 2 C4T S.r.l., Colosseum Combinatorial Chemistry Center, 00133 Rome, Italy; 3 Structural Biology and Biophysics Unit, Fondazione Ri.MED, 90100 Palermo, Italy; 4 Target Identification and Screening Group, Fondazione Ri.MED, 90100 Palermo, Italy; 5 Medicinal Chemistry Group, Fondazione Ri.MED, 90100 Palermo, Italy; 6 Axxam SpA, 20091 Bresso, MI Italy; 7 Dipartimento di Medicina di Precisione, Università degli Studi della Campania “L. Vanvitelli”, 80100 Naples, Italy; 8 Department of Pathology and Medical Biology, University Medical Center Groningen, University of Groningen, 9713 Groningen, GZ The Netherlands; 9 Advanced Data Analysis Group, Fondazione Ri.MED, 90100 Palermo, Italy; 10 BIOGEM, 83031 Ariano Irpino, AV Italy; 11 IEOS-CNR, 80100 Naples, Italy

**Keywords:** Epigenetic regulation, Lysine demethylases, KDM4 inhibitors, Fragment-based drug discovery (FBDD), KDM4A, Rational drug design

## Abstract

**Background:**

Lysine demethylase enzymes (KDMs) are an emerging class of therapeutic targets, that catalyse the removal of methyl marks from histone lysine residues regulating chromatin structure and gene expression. KDM4A isoform plays an important role in the epigenetic dysregulation in various cancers and is linked to aggressive disease and poor clinical outcomes. Despite several efforts, the KDM4 family lacks successful specific molecular inhibitors.

**Results:**

Herein, starting from a structure-based fragments virtual screening campaign we developed a synergic framework as a guide to rationally design efficient KDM4A inhibitors. Commercial libraries were used to create a fragments collection and perform a virtual screening campaign combining docking and pharmacophore approaches. The most promising compounds were tested in-vitro by a Homogeneous Time-Resolved Fluorescence-based assay developed for identifying selective substrate-competitive inhibitors by means of inhibition of H3K9me3 peptide demethylation. 2-(methylcarbamoyl)isonicotinic acid was identified as a preliminary active fragment, displaying inhibition of KDM4A enzymatic activity. Its chemical exploration was deeply investigated by computational and experimental approaches which allowed a rational fragment growing process. The in-silico studies guided the development of derivatives designed as expansion of the primary fragment hit and provided further knowledge on the structure–activity relationship.

**Conclusions:**

Our study describes useful insights into key ligand-KDM4A protein interaction and provides structural features for the development of successful selective KDM4A inhibitors.

**Supplementary Information:**

The online version contains supplementary material available at 10.1186/s13148-023-01613-7.

## Background

Epigenetic regulation plays a crucial role in maintaining the unique cellular identity and is involved in biological processes such as proliferation, development, differentiation, and genome integrity [1, 2]. Reversible methylation of lysine residues in histone proteins is one of the most prominent epigenetic mechanisms and is regulated by the interplay of lysine methyltransferases (KMTs) and demethylases (KDMs) [3].

Among demethylases, the Jumonji C (JmjC) domain-containing family [4–7] acts through an oxidative mechanism using Fe(II) and *α*-ketoglutarate (*α*-KG or 2-OG) as cofactors [8]. In humans, around 20 histone demethylases containing the JmjC domain have been identified and, based on the structural homology, they are clustered into different subfamilies (KDM1-8) [9, 10]. The *N*-methylation of histone lysine is crucial for gene transcription modulation and can lead to different effects on the chromatin state and to several functional outcomes depending on the specific methyl-substrate recognized by the protein and on the number of methyl marks involved.

The KDM4 subfamily shows functional diversity due to the variability in the substrate specificities [11–14]. The demethylation of histone-3-lysine-36 is generally associated with splicing and active transcription [4], while demethylation of histone-3-lysine-9 is linked to gene silencing [7]. More specifically, the isoforms KDM4A-B-C can efficiently demethylate the tri- and di-methylated forms of both histone H3 lysine 9 (H3K9me3/me2) and lysine 36 (H3K36me3/me2), whereas KDM4D is more efficient at H3K9me2 than H3K9me3 but is unable to recognize H3K36me3 [4, 14, 15]. Depending on the specific gene expression pathway involved, the gene amplification and overexpression of KDM4 proteins can lead to different biological outcomes, such as chromosomal stability change, inactivation of tumor suppressors, promotion of oncogene expression, hormone receptor binding and downstream signaling [16–18]. Of note, deregulation of KDM4A isoform has been correlated to carcinogenesis and tumor progression and has been observed in breast [19–23], prostate [7, 24–27], lung [28, 29], colon [30], endometrial [31–33] and bladder [34] tumours. These findings have made KDM4A an attracting target for cancer therapeutics. [35]

**Fig. 1:**
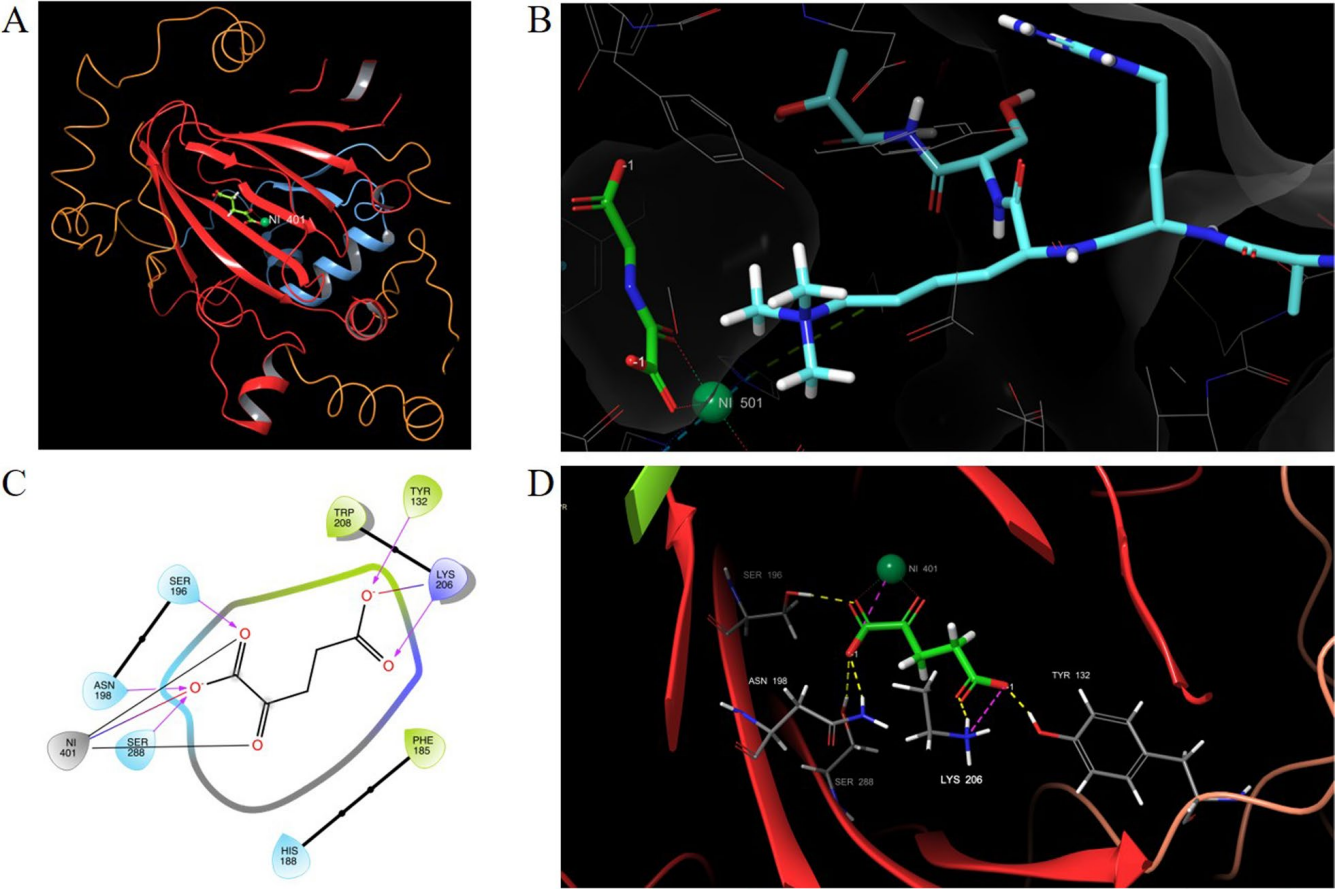
Structural details of the N-terminal region of KDM4A (JMJD2A). **A** Crystal structure of KDM4A (JMJD2A) in complex with 2-OG (PDB 5TVR), which shows a close interaction between the JmjC domain and the JmjN domain, in red and light blue, respectively. **B** Crystal structure of JMJD2A complexed with histone H3 peptide trimethylated at Lys9 (PDB code: 2OQ6). 2-OG is shown in green sticks, H3 peptide in light blue sticks, and Nickel ion in green sphere. The histone substrate is bound in a broad hydrophobic cleft by adopting a bent conformation with the methylated nitrogen of the lysine side chain oriented towards the metal ion, in order to enable the catalytic reaction. **C** and **D** Detailed view of 2-OG binding mode in 2D and 3D, respectively. The hydrogen bonds are depicted in yellow and electrostatic interactions in magenta. 2-OG interacts through a salt bridge with Lys 206, and H-bonds with both Lys206 and Tyr132

KDM4A protein comprises a catalytic core formed by JmjN domain, JmjC domain and a zinc finger motif, and contains double non-catalytic domains as two plant homeodomains (PHD), and two Tudor domains (Fig. 1) [36]. The JmjC domain is the catalytic center and interacts widely with the close JmjN domain, which is essential to maintain structural integrity. Furthermore, the zinc ion plays a key structural role for the formation of an active catalytic core [37], while the non-catalytic domains are involved in several functions including substrate specificity and regulation of the enzyme activity [38, 39]. The histone substrates are bound in a broad hydrophobic cleft of the lysine binding site by adopting a bent conformation with the methylated nitrogen of the lysine side chain oriented in a deep catalytic pocket toward the Fe(II) ion, in order to enable the catalytic reaction (Fig. 1B). The crystal structure of KDM4A in complex with the natural cofactor *α*-KG, shows that *α*-KG interacts through a salt bridge with the basic residue Lys 206 and forms H-bond interactions with the side-chain of Lys206 and Tyr132 H-bond donor residues (Fig. 1C–D). [40–43]

To date, several classes of KDM4 inhibitors with different mechanisms of action have been identified [44, 45], and the most potent compounds seem to act via a competitive mechanism by displacing the *α*-KG [46]. However, none of these compounds made to the market due to lack of selectivity, although some entered the clinical studies phase. [45]

In this scenario, in order to identify new KDM4A inhibitors, we decided to perform a fragment-based framework, starting from a structure-based virtual screening (VS) campaign. Fragment-based screenings are indeed well recognized as valuable approaches, especially for hard-to-drug targets. Compared to small molecules, fragments show more ‘atom-efficient’ binding interactions, therefore they can be considered as a more efficient starting point for subsequent optimization [47]. Fragment-based approach, could be therefore helpful to overcome selectivity issues, which are typical of KDM4, in later phases of hit optimization and hit-to-lead-optimization.

A collection of fragments was extracted from commercial libraries and screened using docking and structure-based pharmacophore approaches. Based on the computational outcomes, 225 fragments were selected, purchased, and tested in vitro by a Homogeneous Time-Resolved Fluorescence (HTRF)-based assay developed for identifying selective substrate-competitive inhibitors by means of inhibition of H3K9me3 peptide demethylation. The biochemical screening led to the identification of one preliminary active fragment, for which we conducted an extensive exploration of the binding mode through a computational analysis of selected analogues. This in-depth analysis allowed the identification of the key interactions with KDM4A and the subsequent design and synthesis of a new small set of derivatives. All computational outcomes were validated by in-vitro tests aimed at determining both the inhibition potency (IC_50_) and the binding affinity (*K*_*D*_) for each compound. Herein, our study delivers additional guidance for the discovery of new efficient KDM4A inhibitors.

## Results

### Virtual screening campaign led to 225 compounds potentially able to inhibit KDM4A

**Fig. 2:**
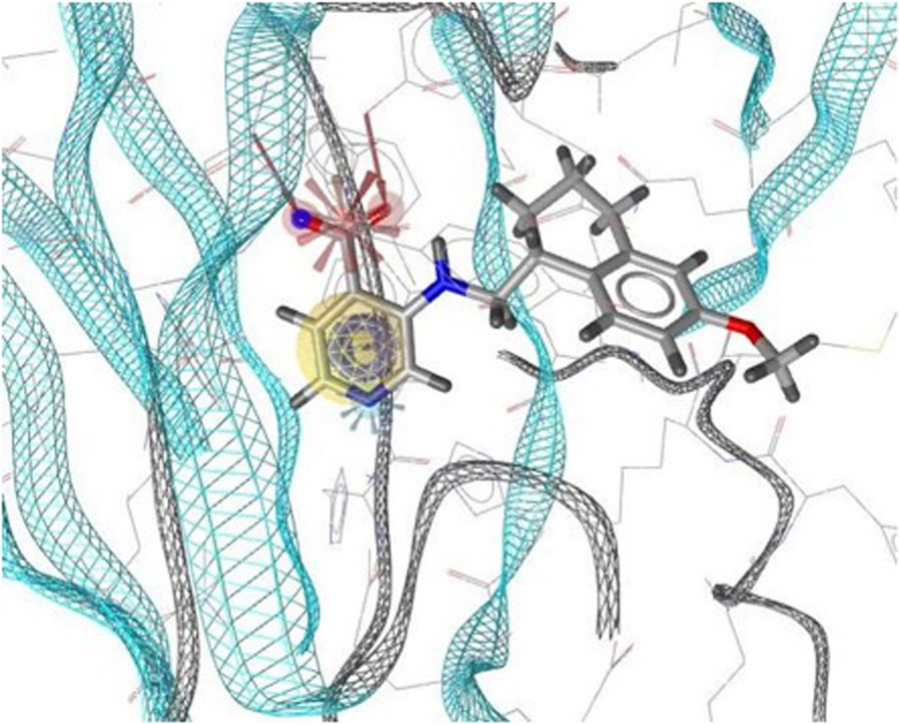
Structure-based pharmacophore model generated on PDB 5VMP. Red arrows represent H-bond acceptors with the red star indicating a negatively charged residue. Yellow sphere refers to the hydrophobic interaction, and in this case overlaps with the aromatic feature in blue. The cyan star represents the metal binding site

With the aim of identifying fragments potentially able to inhibit KDM4A, a virtual screening campaign was performed. Firstly, a rational construction of the virtual fragments’ library was carried out. The fragments were selected from several commercial libraries, applying filters such as the rule of three, which has been proven to be a useful rule for the construction of fragments’ libraries for lead generation [48, 49]. Initially, Pan-Assay INterference compounds (PAINS) and Rapid Elimination Of Swills (REOS) compounds were filtered off, to discard all species that could result as false positives due to the intrinsic reactivity or assay interferences [50]. Then, in order to collect the most suitable fragments, only compounds with molecular weight in the range of 140–300 Dalton and at least one ring system were considered. Furthermore, the physicochemical properties were evaluated, and molecules not aligned to the rule of three parameters were discarded. In addition, the chemical space was investigated to avoid duplicates in the library. As a result, a virtual fragments’ library of 250,000 compounds was obtained, and used to perform the VS campaign to select the most promising fragments to be tested in vitro by biochemical assay. The VS campaign included both pharmacophore and docking approaches. The pharmacophore model was created using LigandScout software [51] starting from the crystal structure of KDM4A protein in complex with the known inhibitor QC5714 (PDB 5VMP) [52]. This structure was chosen because best performed for the creation of our structure-based pharmacophore. In fact, compared to other available structures, it allowed the detection of all the crucial features for the optimal interactions between ligand and protein. Moreover, the pharmacophore model generated from 5VMP resulted the best in terms of AUC and EF values from the ROC curves parameters (Additional file 1: Fig. S1). The newly generated pharmacophore model showed five features at the central core of the QC5714 molecule, including two H-bond acceptors, two positional features in the aromatic pyridine ring (hydrophobic and pi–pi) and a metal chelator feature formed by the nitrogen atom of the pyridine moiety (Fig. 2). Then, according to the pharmacophore-fit score, the best fragments were selected.

In order to perform a more reliable VS campaign, molecules from the virtual fragments’ library were also docked. The docking grid was generated using the crystallographic complex of the protein KDM4A with QC6352 (PDB: 5VGI) [52], and the features of the pharmacophore model were set as constraints, such as Tyr132 H-bond acceptor, Lys206 H-bond acceptor, positional constraint, and metal chelator binding. The docking screening was initially carried out in high-throughput virtual screening (HTVS) mode, retrieving the first 10,000 best ranked fragments. The crystal structure 5VGI well performed for cognate docking results, obtaining a Root-Mean-Square Deviation (RMSD) value of 0.5813. Compounds were thus selected in a consensus mode between the two approaches according to the constraints found within the binding mode, the docking score and the pharmacophore fit score. The use of two different PDB structures for the virtual screening campaign allowed us to minimise the bias of a single “induced-fit’ conformation of the protein.

Subsequently, the best ranked fragments were docked in standard precision (SP) mode in the same model and only compounds matching at least 2 constraint features were retrieved. Finally, the pharmacophore and docking screening data were merged leading to 225 refined compounds potentially able to inhibit KDM4A. The 225 fragments could be clustered in 25 groups based on their chemotype similarity (Additional file 1: Table S1). This ample variability of scaffolds well demonstrates that there is no bias in the used virtual screening approach toward a specific chemotype.

### 2-(Methylcarbamoyl)isonicotinic acid inhibits KDM4A enzymatic activity

**Fig. 3:**
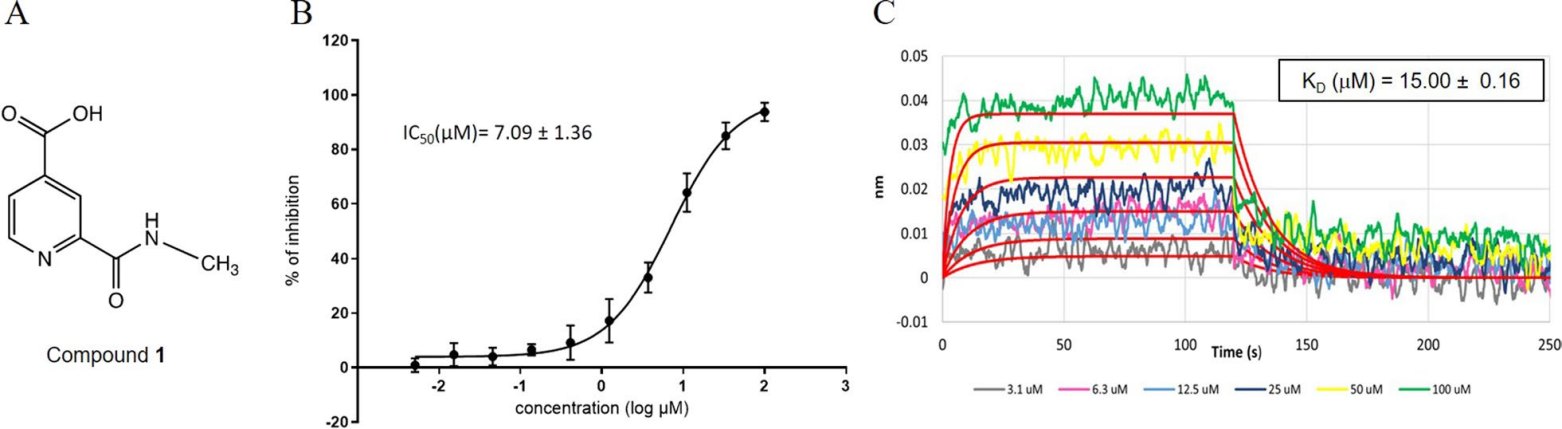
Activity of 2-(methylcarbamoyl)isonicotinic acid in in-vitro assays. **A** Structure of 2-(methylcarbamoyl)isonicotinic acid, namely compound **1**. **B** Dose–response curve of compound **1** against KDM4A. Graph depicts mean and standard deviation of six replicates of the obtained percentage of inhibition at each concentration. Data were fitted using a non-linear 4-parameter logistic model. The obtained IC_50_ ± its standard error is reported as result of the fitting. Analysis was performed by GraphPad Prism 9.0 software (GraphPad Software, Inc., San Diego, CA, United States). **C** BLI sensorgrams for compound **1** against KDM4A. Binding constant (*K*_*D*_) value was estimated by globally fitting the BLI response intensity (nm) as a function of compound concentration (μM) with the Octet Data Analysis Software, using a 1:1 Langmuir binding model

Fragments selected by VS were tested in vitro using a HTRF-based assay [53] developed for identifying substrate-competitive inhibitors by means of inhibition of H3K9me3 peptide demethylation. Recombinant KDM4A used for the HTRF-based assay was prepared in house. Propaedeutically, folding and stability of recombinant KDM4A were tested by far-UV Circular Dichroism (CD) and Proton Nuclear Magnetic Resonance (^1^H NMR), which indicated that our recombinant enzyme remains folded and stable in our experimental conditions (Additional file 1: Fig. S2). Further confirmation of functional recombinant KDM4A was obtained by testing its demethylase activity on H3K9me3 peptide in presence of 2-OG substrate (Additional file 1: Fig. S3). The HTRF-based screening was then performed testing compounds at both 25 and 50 μM, and resulted in 2-(methylcarbamoyl)isonicotinic acid, hereafter compound **1**, as the only active compound out of the 225 selected by VS. The biological activity of compound **1** was further confirmed by a dose–response curve, which resulted in an IC_50_ value of 7.09 ± 1.36 μM (Fig. 3B).

Bio-layer interferometry (BLI) was then used to determine the equilibrium dissociation constant (*K*_*D*_) for the binding event. QC6352 inhibitor [52] and *N*-[(2-Chloro-6-fluorophenyl)methyl]-2-(2,5-dioxo-4-phenyl-4-propylimidazolidin-1-yl)-*N*-methylacetamide [54] were used as positive and negative control, respectively. Biotinylated KDM4A folding and stability was comparable to the unlabelled protein, and DMSO was tolerated up to 5% (Additional file 1: Fig. S4). The interaction of KDM4A with compound **1** resulted in a *K*_*D*_ of 15.00 ± 0.16 μM (Fig. 3C). Binding was also confirmed by Saturation-Transfer Difference (STD) NMR experiments (Additional file 1: Fig. S5).

**Fig. 4:**
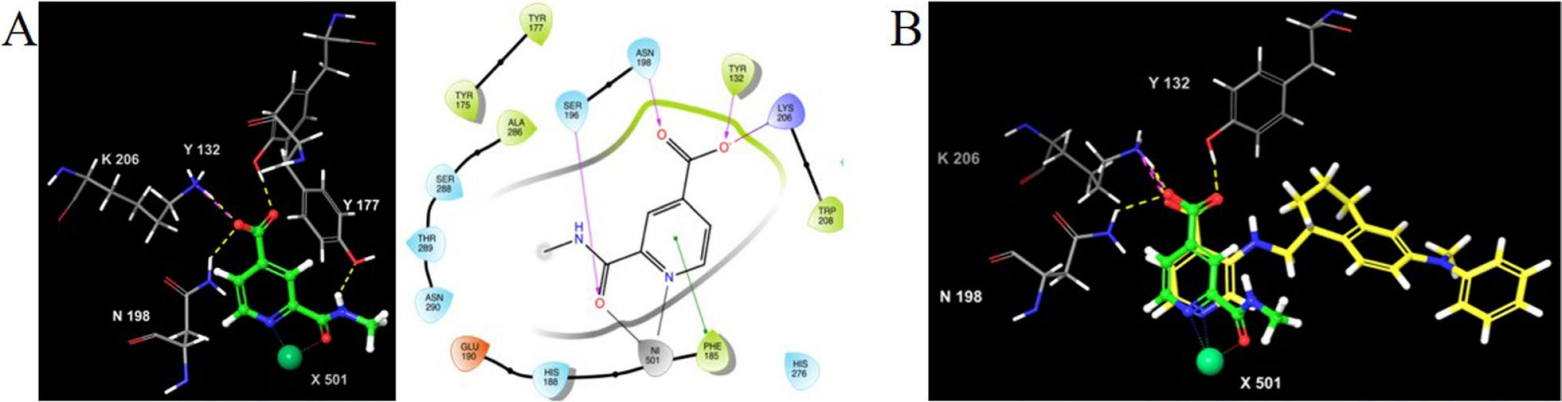
Comparison of the binding pose of compound 1 and lead compound QC6352. **A** Compound 1 binding pose retrieved from Induced-fit docking with 2D ligand–protein interaction. In the picture, pyridine moiety establishes a pi–pi interaction with Phe185. The metal ion is linked to pyridine nitrogen atom and carbonyl group in a bidentate manner. Moreover, the 4- carboxylic acid interacts through a salt bridge with Lys 206, and H-bonds with Tyr 132 and ASN 198. **B** Superimposition of QC6352, in yellow, and compound 1, in pink, in complex with KDM4A

The binding pose of compound **1** was then compared to that of QC6352 when in complex with KDM4A [52], in order to evaluate possible position deviation of the fragment within the binding site. A structure-based analysis of the local environment around the binding pocket was exploited, and docking screening in extra precision mode was carried out. Moreover, induced-fit docking was applied to better explore protein side chains conformational changes depending on fragment decorations [55, 56]. The fragment was correctly posed by the algorithm, and was able to reproduce in a stable manner the central core position observed for the lead compound QC6352 (Fig. 4).

### Computational insights of compound 1 binding mode

**Fig. 5:**
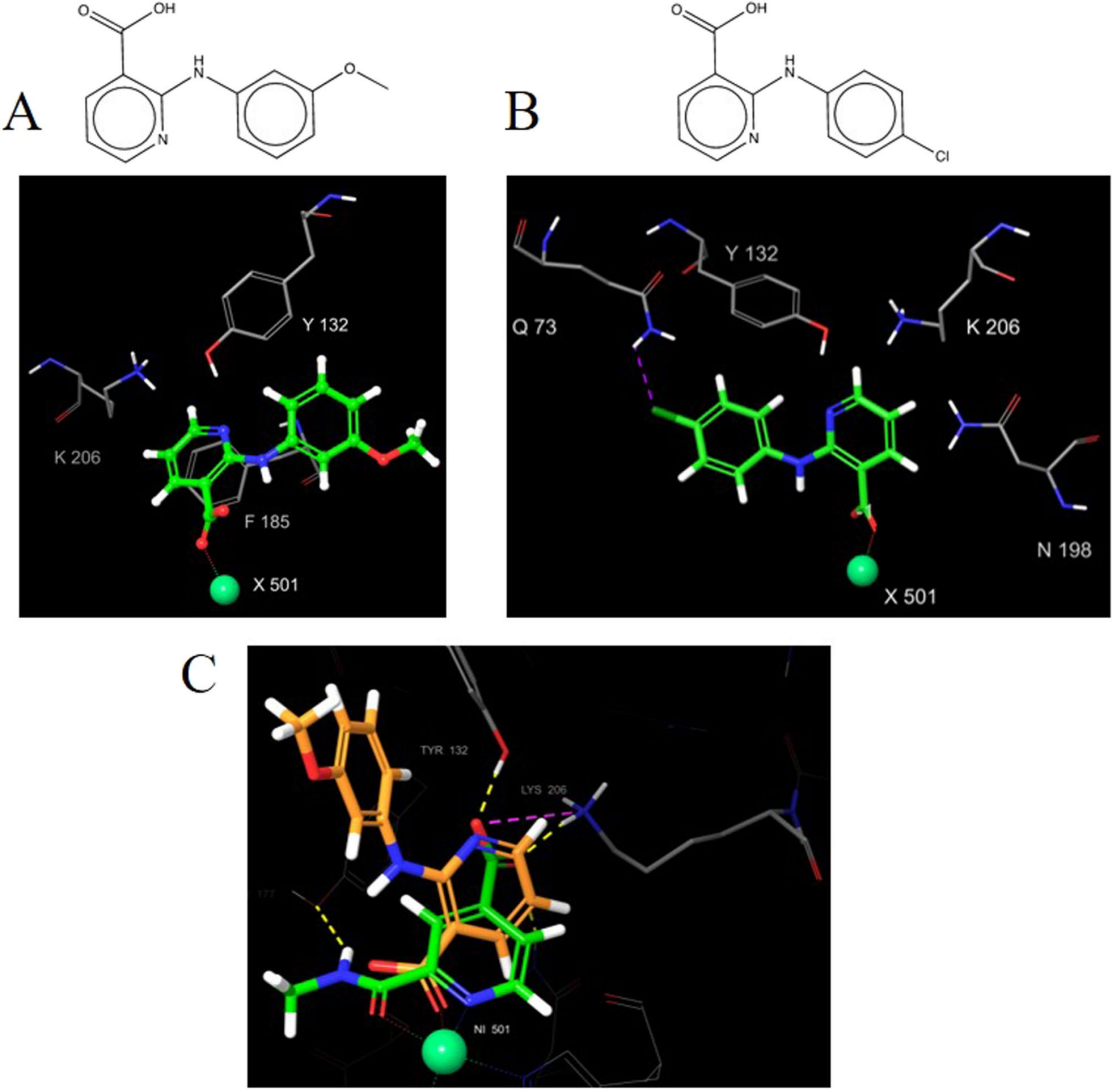
Binding pose of pyridine-3-carboxylic acid derivatives. **A** and **B** structure and binding pose of two selected compound **1** analogues. For both compounds, an amide group and a carboxylic group are present in position 2 and 3, respectively. In figure **A** pyridine moiety is able to establish pi–pi interaction with Phe185 residue and is pointing toward the metal ion, thus retrieving a stable binding pose. Binding pose in figure **B** shows a ligand–protein interaction just through H-bond between carboxylic group and Ser 188. However, these compounds miss interactions with residues Tyr132 and Lys206, known to be crucial in the natural cofactor binding. **C** Overlapping compound **1** and compound shown in (**A**)

With the aim of validating compound **1** chemotype, structural close analogues of this preliminary active fragment were selected. The structural search was carried out in PubChem databases [57], using PubChem fingerprint, and applying a Tanimoto similarity coefficient of 0.9. Analogues variously decorated at the C-2, C-3 and C-4 positions of the pyridine ring were chosen with the aim of exploring how the substituents could affect the binding mode within the catalytic site of KDM4A. Docking studies were then performed and, for each putative binding pose, the interaction scores per-residue were calculated to evaluate the contribution of each H-bond interaction and the internal energy *E*_int_ (Additional file 1: Table S2). The obtained values suggested a favourable interaction when an amide group was present at the pyridine C-2 position. In this case, a favourable *E*_int_ value with Lys 206 and with the metal ion of the catalytic site were observed, thus indicating that this moiety could help the protein–ligand complex stabilisation, probably due to the bidentate chelation. Moreover, we explored in-silico if the introduction of a carboxylic group at the C-3 position could affect the binding mode due to a change in the distance between this group and the pyridine nitrogen. Our docking studies showed that compounds with the carboxylic group at the C-3 position and bearing different amines at the C-2 position, formed a stable complex but missed interactions with residues Tyr132 and Lys206, known to be crucial in the natural cofactor binding (Fig. 5).

In light of these evidences, we decided to focus the chemical exploration on pyridine C-4-carboxylic acid derivatives and bearing substituents at the C-2 position.

### Compound 1 binding mode explored through fragment growth

**Fig. 6:**
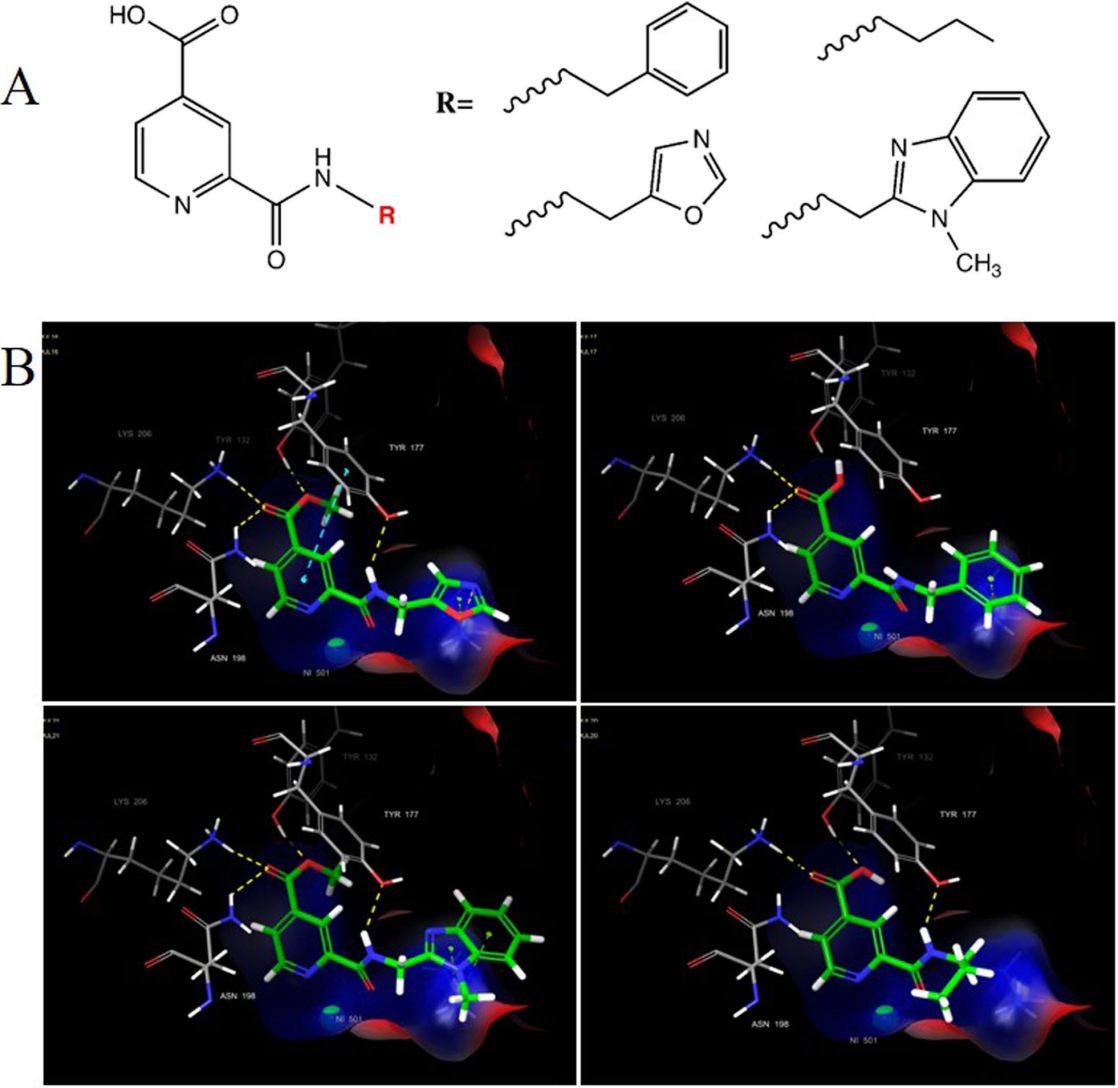
Binding pose of compound 1 derivatives. **A** General structure of compound 1 analogues. **B** Binding poses of some of the most promising compounds newly designed. H-bonds and pi–pi interactions are depicted as yellow and light green dashed lines respectively

With the aim of growing the molecular structure of compound **1** to improve its binding strength and biological activity for KDM4A, we decided to modify the carbamoyl group, keeping in mind the crucial role of the C-2 amide moiety for key interactions with the metal ion. For this purpose, compound **1** derivatives were designed by introducing different amides at the C-2 position, and the binding mode was analyzed by docking (Fig. 6). The most promising analogues exhibited interactions with key residues such as Lys206 and Tyr 132, through the pyridine moiety, whereas the hydrophobic moiety was able to establish pi-pi interactions with crucial aromatic amino acids within the binding pocket (Fig. 6B). Furthermore, these compounds showed interesting *E*_int_ values with the metal ion and with Lys206, suggesting a pivotal retention of the main ligand–protein interactions (Additional file 1: Table S2). Hence, we decided to synthetize this small set of analogues, and evaluate the inhibition activity toward KDM4A.

**Fig. 7:**
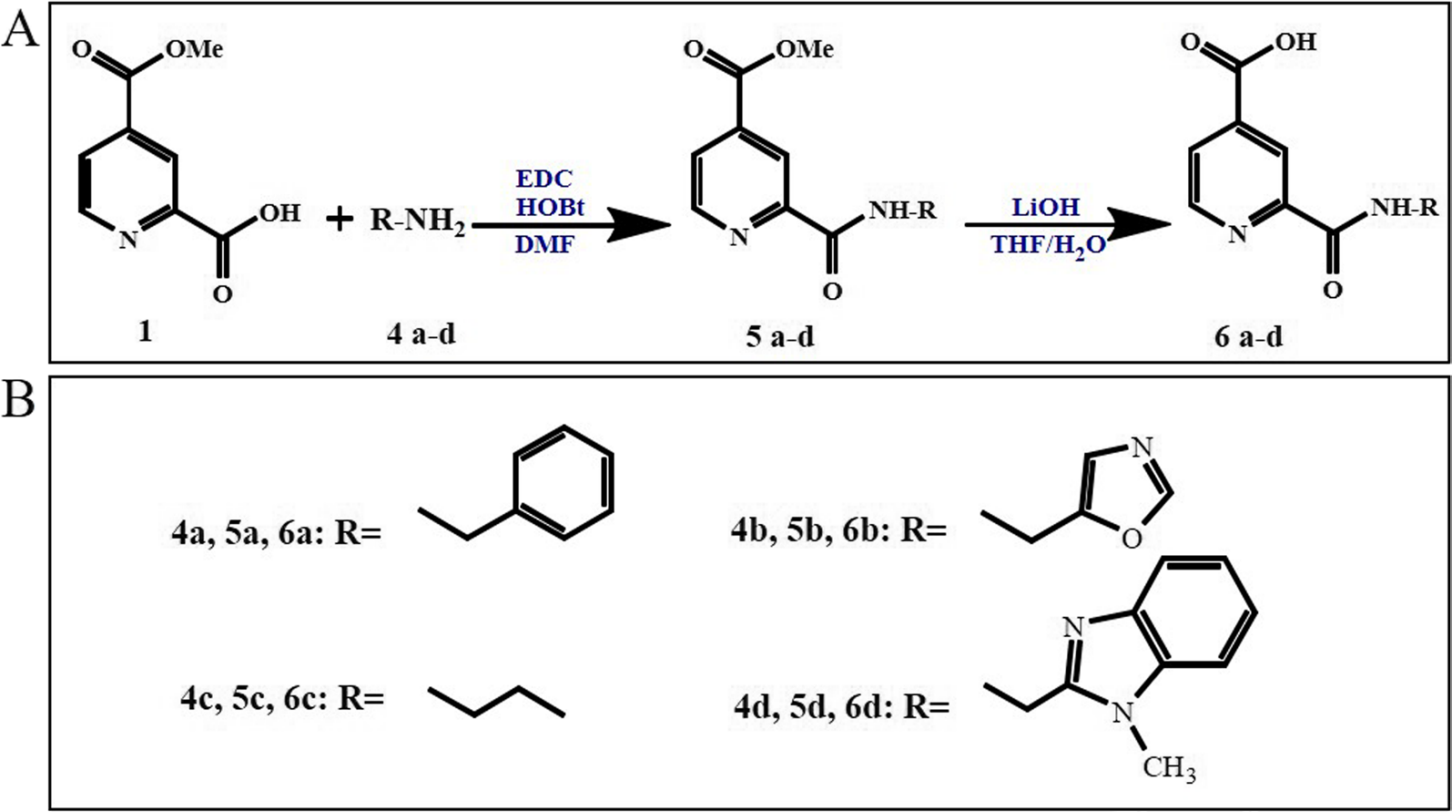
Synthesis of 2-Carbamoylpyridine-4-carboxylic acid derivatives. **A** General scheme of the chemical reactions. Reagents and conditions are indicated. **B** Substituents used as R in 4a-d, 5a-d, and 6a-d

For the synthesis of **5a-d** and **6a-d** derivatives*,* testing compounds were prepared following the synthetic route shown in Fig. 7. For the coupling reaction between the commercial 4-(methoxycarbonyl) picolinic acid (**1**) and the amines (**4a-d**), 1-ethyl-3-(3-dimethylaminopropyl)carbodiimide (EDCI) and hydroxybenzotriazol (HOBt) were used as activating reagents, affording the amides **5a-d**. Hydrolysis with lithium hydroxide gave the carboxylic acid derivative **6a-d**.

**Fig. 8:**
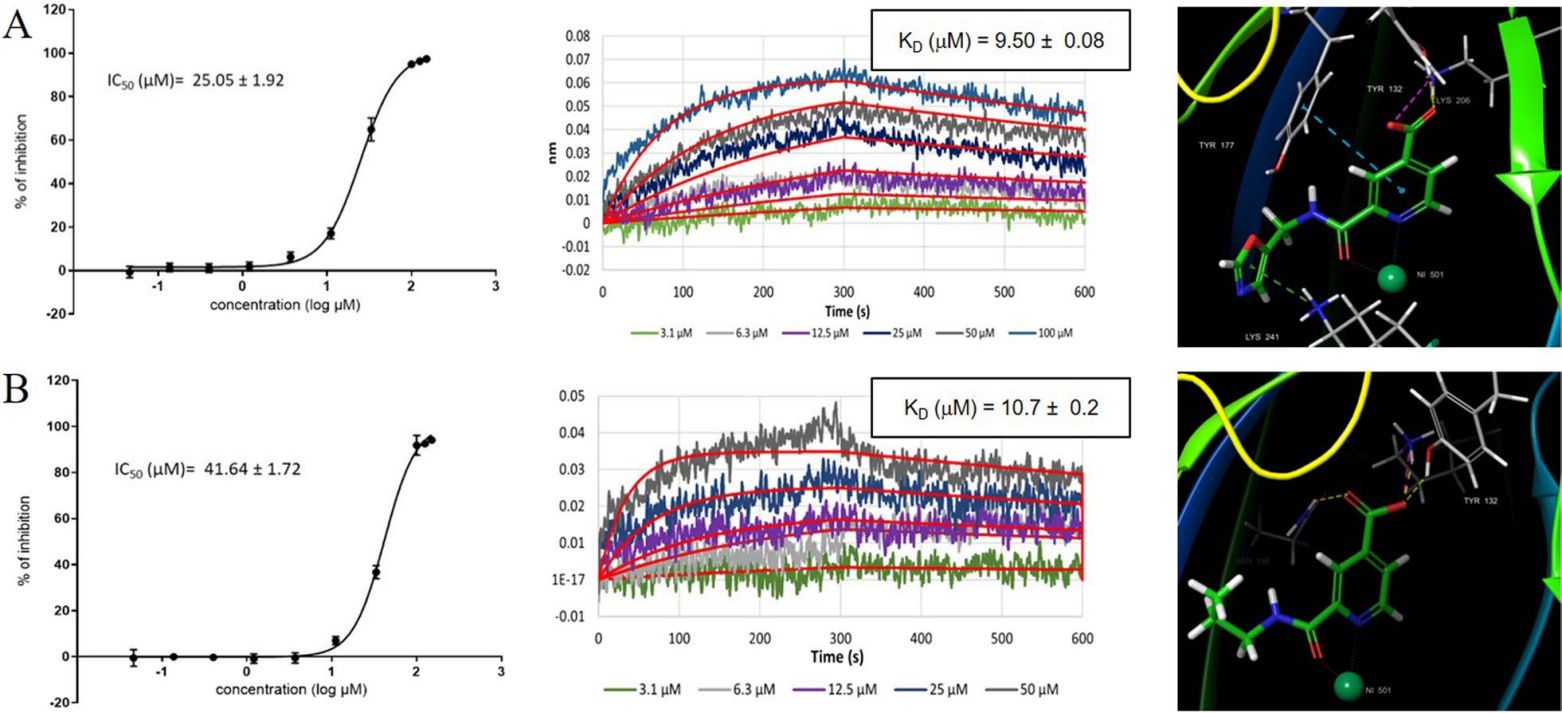
2-Carbamoylpyridine-4-carboxylic acid derivatives 6b and 6c. Dose–response curve (*left*), BLI sensorgram (*middle*), and binding pose (*right*) for compound **6b A**, and compound **6c B**. In the binding pose, H-bond, salt-bridge, and pi-pi interactions are depicted as yellow, violet, green and blue dashed lines, respectively. Fitting of IC50 and BLI curves was performed as already reported in Fig. 3

The biological activity of derivatives **5d and 6a-d** was then evaluated by the HTRF-based assay**.** Compounds **6b** and **6c** resulted the best analogues of this small series, with inhibition higher than 90% at 50 μM concentration, and IC_50_ values of 25.05 μM and 41.64 μM, respectively (Fig. 8). The introduction of 2-ethyl-1-methyl-1*H*-benzo[*d*]imidazole (**5d, 6d**) and benzyl (**5a**, **6a**) groups was detrimental as compounds resulted inactive (inhibition < 50%, at the concentration of 50 μM).

The binding constant for derivatives **6b** and **6c** was also determined by BLI, resulting in K_D_ values of 9.5 μM and 10.7 μM, respectively (Fig. 8).

Analysis of the predicted binding poses for derivatives **6b** and **6c** showed that these compounds maintained the same key interactions as compound **1**, binding Lys206 and Tyr132 within the 2-OG pocket through the carboxylic acid group and the zinc ion, in a bidentate manner. In addition, the pyridine core of compound **6b** and the oxazole moiety, established pi-pi and pi-cation interactions with Tyr177 and Lys241, respectively (Fig. 8). Lipophilic efficacy LE and LLE scores [58] for **6b** and **6c** compounds were also determined. These values resulted to be in the optimal range, indicating that these compounds are suitable for further optimization.

Values of IC_50_, *K*_*D*_, LE and LLE for derivatives **5a**, **5d, and 6a-d** are summarized in Table 1.

**Table 1:** Values of IC_50_, K_D_, LE and LLE for compound 1 and derivatives (5a, 5d and 6a–d)

Compound ID	Compound structure	IC_50_ (μM)	*K*_*D*_ (μM)	LE	LLE
**1**		7.1 ± 1.4	15.00 ± 0.16	0.51	4.59
**5a**		N.D Inhibition at 50uM < 50%	N.D		
**5d**		N.D Inhibition at 50uM < 50%	55.0 ± 2.0	0.24	1.88
**6a**		N.D Inhibition at 50uM < 50%	45.0 ± 1.0	0.33	2.49
**6b**		25.0 ± 1.9	9.50 ± 0.08	0.39	4.50
**6c**		41.6 ± 1.7	10.7 ± 0.2	0.48	4.18
**6d**		N.D Inhibition at 50uM < 50%	> 50	N.D	N.D

## Discussion

Histone methylation and demethylation balance is essential to maintain cellular homeostasis. Aberrant activity of lysine demethylases is frequently observed in cancer and can result in different biological outcomes, depending on the specific gene expression pathway involved. There are several evidences to suggest that overexpression and activation of KDM4A protein are involved in carcinogenesis and tumor progression, acting as both promoter of oncogenes and negative modulators of onco-suppressor. Therefore, KDM4A may represent a suitable therapeutic target for cancer treatment. To date, several chemotypes of KDMs inhibitors have been identified. Nevertheless, most of these compounds lack selectivity.

In this study, we employed a rational and systematic approach to identify potential inhibitors of KDM4A. Our virtual screening campaign resulted in the identification of 225 compounds with the potential to inhibit KDM4A enzymatic activity. Among these compounds, 2-(methylcarbamoyl)isonicotinic acid, referred to as compound **1**, was found to be the most promising, displaying an inhibition potency of 7.09 ± 1.36 μM for KDM4A.

The success of our VS campaign was facilitated by the construction of a virtual fragments’ library, selected from various commercial libraries based on specific filters. We adhered to the rule of three to ensure the selection of suitable fragments for lead generation. To eliminate false positives, we filtered out Pan-Assay INterference compounds (PAINS) and Rapid Elimination Of Swills (REOS) compounds. Subsequently, we considered only compounds with appropriate molecular weight and possessing at least one ring system. Compounds containing toxicophores or highly reactive groups were excluded as these would be undesirable compounds to use in the development of drug candidates. This process yielded a virtual fragments’ library of 250,000 compounds, which formed the basis for our VS campaign.

To enhance the reliability of our VS results, we adopted a dual approach employing both pharmacophore and docking techniques. The pharmacophore model was constructed based on the crystal structure of KDM4A in complex with the known inhibitor QC5714 (PDB 5VMP), and allowed to get the interaction features map required for optimal interactions between ligand and protein. This analysis enlightens two H-bond acceptors, two positional features in the aromatic ring and a metal chelator feature, needed to interact with the metal ion inside the binding pocket. The features of the pharmacophore model were exploited as constraints in the docking grid, in order to retrieve molecules that maintained the crucial interactions. Docking studies were then performed using the crystallographic complex of KDM4A with QC6352 (PDB: 5VGI). By employing two different protein structures, we minimized potential biases arising from a single "induced-fit" conformation of the protein. Based on consensus between the docking score and the pharmacophore fit score, 225 fragments were finally selected to be tested in vitro using a HTRF-based assay. Notably, the key residues highlighted in this study, were Tyr132, Lys206 as H-bond acceptor, usually involved in the natural cofactor binding and Zn (II) ion for metal chelator binding, engaged in histone substrate positioning process inside the protein.

In vitro testing of the selected fragments revealed that compound **1**, 2-(methylcarbamoyl)isonicotinic acid, was the most promising active compound among the 225 candidates. Further dose–response and binding studies confirmed its inhibitory activity, with an IC_50_ value of 7.09 ± 1.36 μM and a *K*_*D*_ of 15.00 ± 0.16 μM, respectively. The binding pose of compound **1** was in good agreement with the crystallographic structure of KDM4A in complex with QC6352, affirming its stable positioning within the binding site.

To better understand the structural requirements for KDM4A inhibition, we performed computational analysis and explored structurally related analogs of compound **1**. To explore the effect of scaffold substituents on the binding pose/binding activity, compound **1** analogs variously decorated at the C-2, C-3 and C-4 positions of the pyridine ring were selected and analyzed by docking studies. More in detail, our computational investigation highlighted the pivotal role of the carboxylic group at the C-4 position with key interactions with Lys 206 and Tyr 132 residues laying in the catalytic core of KDM4A. We also demonstrated that when the carboxylic group was moved to C-3 position, the binding mode changed, and the biological activity toward KDM4A was lost. Moreover, we proved that the presence of an amide group at the C-2 position with hydrophobic substituents resulted in pi-pi interactions with aromatic residues of the KDM4A binding pocket, and promoted the metal ion chelation in a bidentate manner. This scaffold exploration allowed the rational design and synthesis of a new set of compound **1** close analogues bearing different amides at the pyridine C-2 position of the pyridine ring.

By varying substituents on the pyridine ring, we identified **6b** and **6c** derivatives as the most promising analogs with significant inhibitory activity, with IC_50_ values of 25.05 μM and 41.64 μM, respectively. The binding modes of **6b** and **6c** closely resembled that of compound **1**, reinforcing the importance of key interactions with Lys206 and Tyr132 within the 2-OG pocket and the metal ion. The favorable interactions of **6b** and **6c** with KDM4A were also evident in their *K*_*D*_ values of 9.5 and 10.7 μM, respectively. Moreover, LE and LLE scores for 6b and 6c compounds resulted to be in the optimal range for further structural optimization.

The success of our study lies in the rational design of the virtual fragments’ library, the effective combination of pharmacophore and docking techniques in the VS campaign, and the subsequent experimental validation of compound **1** and its analogs. These compounds hold great potential for future therapeutic applications targeting epigenetic dysregulation in various cancers.

## Conclusions

Herein, a fragments screening campaign was performed with the aim of identifying new KDM4A inhibitors. This work is based on the combination of computational and experimental tools used in a synergistic way to rationally explore the chemical environment of a fragment. All information obtained from the computational analysis were supplemented by in-vitro tests aimed at determining both the inhibition potency (IC_50_) and the binding affinity (*K*_*D*_) for each compound. The main outcomes of this work revealed 2-(methylcarbamoyl)isonicotinic acid, here named compound **1**, as fragment hit and enlightened the key role of the carboxylic group at the C-4 position and of the amide group at the C-2 position, and the importance of the hydrophobic nature of the C-2 amide as demonstrated by results showed by compound **1** and its derivates.

Our findings not only provide valuable insights into the molecular interactions of KDM4A inhibitors but also offer promising starting points for further optimization and the development of more potent and selective KDM4A inhibitors. Further studies are necessary to guide the growth and structural optimization of the preliminary hit compounds obtained in this study.

## Methods

### Creation of fragments virtual screening library

The virtual fragments library was created merging in a unique file several available virtual libraries, including Prestwick, Life Chemical, BioAscent, ChemDiv, Asinex, chemBridge, Discovery Chemistry and Enamine. The libraries were prepared using LigPrep, tool of Maestro program, using OPLS3e as force field, and EpiK to generate all the possible states at pH of 7.4 ± 0.2. Desalt and generated tautomers were flagged on, the chirality was retained and at most 32 conformers per ligand were generated. The compounds physicochemical properties were calculated using Qikprop and all the compounds with ≤ 3 H-bond donors, ≤ 3 H-bond acceptors, molecular weight in the range of 140 < MW < 300 Da, predicted Log P (ClogP) ≤ 3 and polar surface area (PSA) ≤ 60Å^2^ were retained. Then, using Canvas [59, 60], the library was filtered by PAINS and REOS [50] in order to discard possible reactive compounds. In addition, with the aim of avoiding close analogues in our library and ensure a sufficient chemical diversity, a similarity matrix, based on linear fingerprint, was generated and a diversity metric was calculated exhibiting a value of 0.025, indicating a sufficient diversity in the fragment library.

### Pharmacophore model creation and screening

The pharmacophore model was generated starting from the PDB coordinates of the ligand–protein complex (PDB ID: 5VMP) [52], using LigandScout software [51]. In the screening the “pharmacophore fit-score” was employed as scoring function and “match all query features” was applied as screening mode. In order to validate Pharmacophore models, 200 actives were retrieved from CHEMBL [61, 62] according to max IC_50_ Value of 10 µM. DUD-E database [63] was then used to generate 1849 decoys. Both actives and decoys were prepared according to the same protocol used for the virtual screening library (LigPrep tool of Maestro program, using OPLS3e as force field, and EpiK to generate all the possible states at pH of 7.4 ± 0.2. Desalt and generated tautomers were flagged on, the chirality was retained and at most 32 conformers per ligand were generated).

### Protein structure preparation and docking screening

The crystal structure of the protein KDM4A in complex with the ligand QC6362 (PDB ID 5VGI) [52], with a resolution of 2,07 Å, was used for the set-up of our model. *Protein Preparation Wizard* [64] by Schrödinger software was used as tool for the protein preparation, adding bond orders and hydrogens to the crystal structure and deleting waters beyond 5.00 Å. Het states at pH 7.4 ± 0.2 were generated with *Epik* and then the protonation state of the protein and the ligand were optimized using *PropKa* at pH 7.4 [65, 66]. The docking grid was generated using *Glide* software released by Schrödinger (release 2018–4) [67], setting the scaling factor at 1.0 Å with a partial charge cut-off of 0.25, and choosing the ligand to define the grid centroid. The pharmacophore features retrieved, were set in the docking grid as constraints: H-bond acceptor on Tyr132, and Lys206, metal acceptor and positional constraint. The searching algorithm on our model was tested using a cognate docking of the co-crystallized ligand, and we obtained an RMSD value of 0.5813. The virtual fragments library was screened in HTVS mode, limiting the number of poses to report at 10,000 compounds. Then, these 10,000 fragments were docked in standard precision mode. For the optimization study, in order to deepen the binding mode of compound **1**, the fragment docking was performed using the extra precision (XP) protocol. In the docking screenings, OPLS3e was used as force field [68] and the van der Waals radii scaling factor was set as 0.8, with a partial charge cut off by 0.15. The ligands were considered as flexible, and Epik state penalties were included to docking score.

The induced-fit docking was performed employing a standard protocol, generating up to 20 poses. Ring conformations were sampled with an energy window of 2.5 kcal/mol and the receptor and ligand van der Waals scaling factors were set at 0.5. The refinement was carried out with Prime and the redocking was performed in extra precision mode.

### Expression and purification of recombinant KDM4A

The plasmid pGEX4T2-JMJD2A encoding human KDM4A residues 1–350 with a GST-6xHis tag at the N-terminal, followed by Tobacco Etch Virus (TEV) cleavage site, was used for protein expression. The plasmid was used to transform T7 Express *E.coli* competent cells. Cells were grown in LB media supplemented with ampicillin (100 µg/mL) at 37 °C until the OD600 reached a value around 0.4. Flasks were then cooled down and, after induction by 0.2 mM ITPG at OD_600_ = 0.8, bacteria were grown at 18 °C for 20 h. After centrifugation at 8000 g (20 min at 4 °C) the obtained pellets were suspended in lysis buffer (20 mM TRIS pH = 8, 0.3 M NaCl, 10 mM imidazole, 5% glycerol, 1 mM DTT, 10 µg/mL DNAse, 0.5 mg/mL lysozyme, 0.5 mM PMSF, 2 cOmplete™ EDTA-free Protease Inhibitor Cocktail, 5 mM MgCl_2_) and incubated at 25 °C for 1 h. Sonication (1 s ON and 1 s OFF) on ice for 7 min at 48% amplitude was performed. 0.1% Triton X-100 was added and the lysate was incubated on ice for 15 min in agitation. Soluble and insoluble phase were separated by centrifugation at 15,000 rpm, 4 °C for 30 min. Soluble phase was filtered using a 0.45 um syringe filter and directly loaded in 2 × 5 mL His-trap FF Crude column (GE Healthcare) previously equilibrated with loading buffer (20 mM TRIS, pH = 8, 0.3 M NaCl, 1 mM DTT, 10 mM imidazole, 5% glycerol). A linear gradient elution by 500 mM imidazole was done to elute bound KDM4A. In order to remove the GST-6xHis tag and imidazole excess, the eluted KDM4A was incubated with His-tagged TEV-protease (molar ratio 20:1) and dialyzed against 20 mM TRIS, pH = pH 7.5, 0.3 M NaCl, 1 mM DTT overnight at 4 °C. Tag free KDM4A was recovered reloading the dialyzed sample onto a His-trap column and collecting the flow-through. Size exclusion chromatography was used as final polishing step using a Superdex 200 grade 16/600 pg column (GE Healthcare) pre-equilibrated with 10 mM Hepes, pH = 7.5, 150 mM NaCl, 5% glycerol, 0.5 mM TCEP. Fractions containing KDM4A were pooled and concentrated to 50–100 µM, flash frozen in liquid nitrogen, and stored at − 80 °C. Protein concentration was measured by using the predicted molar extinction coefficient (*ε*_280_ = 73,800 M^−1^ cm^−1^), and the purity verified by SDS-PAGE resulted > 95%.

### Circular dichroism measurements

Secondary structure of KDM4A and thermal denaturation profiles were investigated by using a Jasco J-1500 spectropolarimeter equipped with a Peltier type cell holder. Samples were prepared using a KDM4A concentration of 20 μM in either 10 mM Hepes, pH = 7.5, 0.5 mM TCEP, or 20 mM NaPh, pH = 7.5, 0.5 mM TCEP, with varying concentrations of NaCl in both buffer’s conditions (0 mM, 75 mM and 150 mM). Far-UV spectra were recorded in 0.01 cm quartz cuvette from 260-200 nm at 25 °C and 37 °C, using a scan speed of 50 nm/min, a 1 nm bandwidth, and a data pitch of 0.5 nm. Each spectrum is the result of 10 accumulations. Baseline correction was obtained by subtraction of the appropriate buffer spectrum. Spectra of biotinylated KDM4A at 25 °C in 10 mM Hepes, pH = 7.5, 150 mM NaCl, 0.5 mM TCEP were also collected. Thermal unfolding curves were obtained by monitoring the ellipticity at 222 nm in the temperature range 20–95 °C (heating rate 2 °C/min).

### Enzymatic HTRF-based assay

Enzymatic HTRF-based assay was run in two steps: enzymatic reaction and detection. Optimal conditions for both steps were determined by considering: plate format, time and temperature of incubation, substrate and DMSO concentrations.

The assay was validated following NIH guidelines [69], and the robustness was proven by determining the *Z*’ value [70], which resulted > 0.5 for all tested screening plates.

The enzymatic reaction mixture (final volume 40 µl) was prepared in 50 mM HEPES pH 7.5, 0.01% BSA, 0.01% Tween20, such to contain 400 nM recombinant KDM4A, 0.5 µM *α*-Ketoglutaric Acid Monopotassium (2-OG, Sigma Aldrich K2000), 300 nm Histone H3(1–21) lysine 9 tri-methylated biotinylated peptide (AnaSpec AS-64360), 5 µM ammonium iron (II) sulfate hexa-hydrate (Sigma Aldrich 215,406) and 1 mM sodium ascorbate (Sigma Aldrich 1140). Dimethyl sulfoxide (DMSO, Sigma-Aldrich D58791) final concentration was 1%. The reaction mixture was incubated for 2 h at 20 °C in ½ area 96 well plates (ref. 6002290 m, Perkin Elmer).

For the detection step, 40 μl of HTRF reagents, H3K9 me2-Eu(K) Ab (Cisbio Bioassays, 61KB2KAE) and Streptavidin XL-665 (Cisbio Bioassays, 610SAXLA), prepared in detection buffer following manufacturer’s instruction (Cisbio Assays, 62SDBRDD), were added to each well and incubated o/n at 20 °C. Fluorescence was measured with a TECAN Spark reader (Männedorf, Switzerland), by setting excitation wavelength at 320 nm and emission wavelength at 620 and 665 nm.

Automation was performed using Bravo (Agilent) liquid handling system. Validation of the assay was performed following NIH guidelines. 2,4-pyridinedicarboxylic acid monohydrate (2,4-PDCA, Sigma Aldrich P63395) was used as positive control at I_max_ 10 µM, and Z’ was determined on 9 independent experiments always giving a value > 0.5. The following reference compounds were used as internal controls in the screening plates: 2,4-Pyridinedicarboxylic Acid, 8-hydroxyquinoline-5-carboxylic acid, pyrido[3,4-d]pyrimidin-4(3H)-one, 3-(ethylsulfonyl)-N-(4-(pyridin-2-yl)thiazol-2-yl)benzamide, 3-(ethylsulfonyl)-N-(4-(pyridin-2-yl)thiazol-2-yl)benzamide, 3-(methylsulfonyl)-N-(4-(pyridin-2-yl)thiazol-2-yl)benzamide.

Analysis was performed by GraphPad Prism 9.0 software (GraphPad Software, Inc., San Diego, CA, United States).

*Bio-layer Interferometry experiments* Bio-layer Interferometry was employed to determine the equilibrium dissociation constant (*K*_*D*_) for compounds binding to KDM4A, using a BLI Octet RED96e instrument (Fortebio–Sartorius). Biotinylated KDM4A samples (20 μg/ml) in 10 mM Hepes, pH = 7.5, 150 mM NaCl, 0.5 mM TCEP, 5% glycerol, and 5% DMSO, were loaded onto super streptavidin (SSA) coated biosensors for at least 500 s to saturate the sensors, and then quenched with 10 µg/ml of biocytin for 60 s. Loaded SSA biosensors were then equilibrated in buffer for at least 600 s prior to baseline collection. Association was followed for at least 120 s or until plateau was reached, in a concentration range of 15.6–500 nM for the positive control, and of 3–200 µM for the negative control, and compound **1** and its analogues. Dissociation was followed for at least 300 s. All steps were performed at 25 °C with an agitation speed of 1000 rpm. QC6352 inhibitor [52] and N-[(2-chloro-6-fluorophenyl)methyl]-2-(2,5-dioxo-4-phenyl-4-propylimidazolidin-1-yl)-*N*-methylacetamide [54] were used as positive and negative control, respectively. Two or three independent experiments were performed for each tested compound.

*K*_*D*_ values were estimated by globally fitting the BLI response intensity (nm) as a function of compound concentration (μM) with the Octet Data Analysis Software, using a 1:1 Langmuir binding model.

Biotinylated KDM4A was obtained as follows. 17 nmol of purified recombinant KDM4A was mixed with 17 nmol of NHS-PEG4-Biotin (ThermoFisher Scientific 21,330) in 10 mM Hepes, pH = 7.5, 150 mM NaCl, 0.5 mM TCEP, 5% Glycerol. The solution was incubated at 4 °C for 2 h. Excess biotin was removed by 2 ml Zeba™ Spin Desalting Columns (7 K MWCO).

*STD-NMR experiments* STD-NMR spectra were recorded at 800 MHz on Bruker spectrometer and 298 K in 20 mM NaPh, pH = 7.5, 150 mM NaCl, 0.5 mM TCEP, 8% D_2_O, using 7.5 µM of KDM4A and 20- or 50-fold excess of each compound. A series of Gaussian-shaped pulses of 0.025 s each was employed with a total saturation time for the protein envelope of 2 s. Off-resonance frequency of *d* = − 40 ppm and on-resonance frequency of *d* = 0.07 ppm (protein aliphatic signals region) were applied. Spectra were processed and analyzed using Bruker TopSpin software.

### Chemical synthesis of 2-carbamoylpyridine-4-carboxylic acid derivatives

Commercially available starting materials, reagents, and anhydrous solvents were used as supplied. Flash column chromatography was performed using Merck silica gel 60 Å 230–400 mesh particle size. Thin layer chromatography was performed using Merck Millipore TLC silica gel 60 F254 sheets and visualized by UV (254 and 356 nm) iodine, and KMnO_4_.

### *General procedure (1) for the synthesis of amides ****5a-d***

A solution of 4-methoxy-carbonyl pyridine-2-carboxylic acid (1 eq.) in anhydrous DMF was cooled to 0 °C. EDCI-HCl (2 eq.) and HOBt (2 eq.) were added and the reaction mixture was stirred for 1 h at room temperature. Next, the amine (1.5 eq.) and DIPEA (3 eq.) were added and the reaction mixture was stirred at room temperature for 16 h. After completion, the mixture was quenched with water and then extracted with ethyl acetate (× 3). The organic layer was washed several times with brine, dried over Na_2_SO_4_ and concentrated under reduced pressure. The crude material was purified by silica gel column chromatography.

*Methyl 2-(benzylcarbamoyl)isonicotinate (****5a****).* According to general procedure (1), 4-methoxy-carbonyl pyridine-2-carboxylic acid (**1**, 0.100 g, 0.55 mmol) and benzylamine (**4a**, 0.089 g, 0.83 mmol) were reacted. The crude product was purified by silica gel column chromatography using 40% EtOAc in DCM as the eluent, affording the desired product **5a** as white powder (0,110 g, 75%).

^1^H NMR (200 MHz, DMSO): *δ* 9.54 (*s*, 1H), 8.85 (d, *J* = 3.2 Hz, 1H), 8.45 (*s*, 1H), 8.06–8.04 (m, 1H), 7.35–7.25 (*m*, 5H), 4.51 (*d*, *J* = 5 Hz, 2H), 3.93 (*s*, 3H).

^13^C NMR (201 MHz, DMSO): *δ* 164.88, 163.81, 150.67, 148.23, 142.52, 138.92, 128.41, 127.42, 127.00, 124.84, 122.26, 52.65, 42.90.

*Methyl 2-((oxazol-5-ylmethyl)carbamoyl)isonicotinate (****5b****).* According to general procedure (1), 4-methoxy-carbonyl pyridine-2-carboxylic acid (**1**, 0.200 g, 1.10 mmol) and 1,3-oxazol-5yl methanamine (**4b**, 0.162 g, 1.65 mmol) were reacted. The crude product was purified by silica gel column chromatography eluting with 5% MeOH in DCM to provide the desired product (**5b**) as white powder (0.120 g, 42%).

^1^H NMR (200 MHz, DMSO): *δ* 9.53 (*d*, *J* = 4 Hz, 1H), 8.85 (d, *J* = 3.2 Hz, 1H), 8.46 (*d*, *J* = 3.4 Hz, 1H), 8.32 (*d*, *J* = 3.8 Hz, 1H), 8.00 (*s*, 1H), 7.11 ((*d*, *J* = 3 Hz, 1H), 4.58 (*d*, *J* = 3.6 Hz, 2H) 3.5 (*s*, 3H).

^13^C NMR (201 MHz, DMSO): *δ* 170.34, 164.80, 163.83, 151.72, 150.66, 142.05, 129.59, 124.82, 123.71, 122.24, 52.62, 33.97.

*Methyl 2-(propylcarbamoyl)isonicotinate (****5c****).* According to general procedure (1), 4-methoxy-carbonyl pyridine-2-carboxylic acid (**1**, 0.100 g, 0.55 mmol) and propylamine (**4c**, 0.05 g, 0.83 mmol) were reacted. The crude product was purified by silica gel column chromatography eluting with 2% MeOH in DCM to provide the product as white oil (0.03 g, 55%).

^1^H NMR (200 MHz, DMSO): *δ* 8.94 (*t*, *J* = 5.6 Hz, 1H), 8.84 (*d*, *J* = 4.9 Hz, 1H), 8.42 (*d*, *J* = 1.7 Hz, 1H), 7.97 (dd, *J* = 4.9, 1.7 Hz, 1H), 3.80 (*s*, 3 H), 3.25 (td, *J* = 7.2, 5.8 Hz, 2H), 1.55 (h, *J* = 7.3 Hz, 2H), 0.90 (*t*, *J* = 7.4 Hz, 3H).

^13^C NMR (201 MHz, DMSO): *δ* 170.35, 164.89, 150.55, 148.14, 142.83, 124.72, 122.17, 59.76, 52.60, 22.13, 11.44.

*Methyl 2-(((1-methyl-1H-benzo[d]imidazole-2-yl)methyl)carbamoyl)isonicotinate (****5d****).* According to general procedure (1), 4-methoxy-carbonyl pyridine-2-carboxylic acid (**1**, 0.300 g, 1.65 mmol) and 1-methyl-1-H-benzimidazol-2-yl methanamine (**4d**, 0.400 g, 2.48 mmol) were reacted. The crude product was purified by silica gel column chromatography eluting with 2% MeOH in DCM to provide the product as white crystal (0.110 g, 20%).

^1^H NMR (800 MHz, DMSO): *δ* 10.05 (*d*, *J* = 7.7 Hz, 1H), 8.92–8.90 (*m*, 1H), 8.53 (*d*, *J* = 1.7 Hz, 1H), 8.11–8.09 (*m*, 1H), 7.95 (*d*, *J* = 8.2 Hz, 1H), 7.79 (*d*, *J* = 8.0 Hz, 1H), 7.62–7.55 (*m*, 2H), 5.05 (*d*, *J* = 5.3 Hz, 2H), 4.05 (*s*, 3H), 3.93 (*s*, 3H).

^13^C NMR (201 MHz, DMSO**):** *δ* 165.89, 165.10, 150.98, 150.56, 149.36, 141.47, 133.04, 126.49 – 125.14 (m), 124.86, 122.40, 114.67, 112.71, 51.60, 35.45, 31.38.

### *General procedure (2) for the synthesis of analogues6a-d*

Lithium hydroxide (3 eq.) was added to a solution of the starting material **5a-d** (1 eq.) in 1:1 THF:H_2_O. The reaction was stirred at room temperature for 1 h. After completion, the reaction mixture was neutralized with 6 N aq. HCl and extracted with ethyl acetate. The organic layer was dried over Na_2_SO_4_ and concentrated under reduced pressure.

*2-(benzylcarbamoyl)isonicotinic acid (****6a****).* According to general procedure (2), methyl 2-(benzylcarbamoyl)isonicotinate (**5a**, 0.05 g, 0.185 mmol) and LiOH (0.013 g, 0.55 mmol) were reacted. The title compound was obtained as white solid (0.035 g, 75%).

^1^H NMR (200 MHz, DMSO): *δ* 9.54 (*d*, *J* = 2 Hz, 1H), 8.86 (*d*, *J* = 3.6 Hz, 1H), 8.48 (*s*, 1H), 8.02 (d, *J* = 3.2 Hz, 1H), 7.34–7.25 (*m*, 5H), 4.51 (*d*, *J* = 3.6 Hz, 2H).

^13^C NMR (201 MHz, DMSO): *δ* 164.88, 163.81, 150.67, 148.23, 142.52, 138.92, 128.41, 127.42, 127.00, 124.84, 122.26, 42.90.

*2-((Oxazol-5-ylmethyl)carbamoyl)isonicotinic acid (****6b****).* According to general procedure (2), methyl 2-((oxazol-5-ylmethyl)carbamoyl) isonicotinate (**5b**, 0.1 g, 0.38 mmol) and LiOH (0.027 g, 1.15 mmol) were reacted. The crude obtained was washed with diethyl ether providing the desired product as white solid (0.02 g, 20%).

^1^H NMR (200 MHz, DMSO): *δ* 9.53 (d, *J* = 4 Hz, 1H), 8.85 (d, *J* = 3.2 Hz, 1H), 8.46 (*d*, *J* = 3.4 Hz, 1H), 8.32 (*d*, *J* = 3.8 Hz, 1H), 8.00 (*s*, 1H), 7.11 ((*d*, *J* = 3 Hz, 1H), 4.58 (*d*, *J* = 3.6 Hz, 2H).

^13^C NMR (201 MHz, DMSO): *δ* 170.34, 164.80, 163.83, 151.72, 150.66, 142.05, 129.59, 124.82, 123.71, 122.24, 33.97.

*2-(Propylcarbamoyl)isonicotinic acid (****6c****).* According to general procedure (2), methyl 2-(propylcarbamoyl)isonicotinate (**5c**, 0.03 g, 0.135 mmol) and LiOH (0.01 g, 0.40 mmol) were reacted. The crude obtained was re-crystallized from ethanol providing the desired product as white solid (0.015 g, 55%).

^1^H NMR (800 MHz, DMSO): *δ* 8.94 (*t*, *J* = 5.6 Hz, 1H), 8.84 (*d*, *J* = 4.9 Hz, 1H), 8.42 (*d*, *J* = 1.7 Hz, 1H), 7.97 (dd, *J* = 4.9, 1.7 Hz, 1H), 3.25 (td, *J* = 7.2, 5.8 Hz, 2H), 1.55 (*h*, *J* = 7.3 Hz, 2H), 0.90 (*t*, *J* = 7.4 Hz, 3H).

^13^C NMR (201 MHz, DMSO): *δ* 166.18, 164.09, 150.55, 149.43, 143.12, 124.74, 122.28, 41.48, 22.44, 11.75.

*2-(((1-Methyl-1H-benzo[d]midazole-2-yl)methyl)carbamoyl)isonicotinic acid (****6d****).* According to general procedure (2), methyl 2-(((1-methyl-1H-benzo[d]midazole-2-yl)methyl)carbamoyl)isonicotinate (**5d**, 0.035 g, 0.108 mmol) and LiOH (0.08 g, 0.324 mmol) were reacted. The crude obtained was washed with diethyl ether providing the desired product as white solid (0.015 g, 45%).

^1^H NMR (800 MHz, DMSO): *δ* 10.05 (*d*, J = 7.7 Hz, 1H), 8.92–8.90 (*m*, 1H), 8.53 (*d*, J = 1.7 Hz, 1H), 8.11–8.09 (*m*, 1H), 7.95 (*d*, J = 8.2 Hz, 1H), 7.79 (*d*, J = 8.0 Hz, 1H), 7.62–7.55 (*m*, 2H), 5.05 (*d*, J = 5.3 Hz, 2H), 4.05 (*s*, 3H).

^13^C NMR (201 MHz, DMSO): *δ* 165.89, 165.10, 150.98, 150.56, 149.36, 141.47, 133.04, 126.49–125.14 (*m*), 124.86, 122.40, 114.67, 112.71, 35.45, 31.38.

### Supplementary Information

**Additional file 1. ** Supplementary Figures and Tables.

## Data Availability

The datasets used and/or analysed during the current study are available from the corresponding author on reasonable request.

## Abbreviations

^1^H NMR: Proton Nuclear Magnetic Resonance
2-OG: 2-Oxoglutarate
*α*-KG: *α*-Ketoglutarate
BLI: Bio-layer Interferometry
CD: Circular Dichroism
ClogP: Predicted Log P
EDCI: 1-Ethyl-3-(3-Dimethylaminopropyl)carbodiimide
FBDD: Fragment based drug discovery
JmjC: Jumonji C domain
JmjN: Jumonji N domain
H3K36me2: Histone H3 lysine 36 di-methylated
H3K36me3: Histone H3 lysine 36 tri-methylated
H3K9me2: Histone H3 lysine 9 di-methylated
H3K9me3: Histone H3 lysine 9 tri-methylated
HOBt: Hydroxybenzotriazol
HTRF: Homogeneous Time-Resolved Fluorescence
HTVS: High-throughput virtual screening
KMTs: Lysine methyltransferases
KDMs: Lysine demethylases
LE: Ligand efficacy
LLE: Lipophilic ligand efficacy
PAINS: Pan-Assay INterference compounds
PHD: Plant homeodomain
PSA: Polar surface area
REOS: Rapid elimination of swills compounds
RMSD: Root-Mean-Square Deviation
SP: Standard precision
SSA: Super streptavidin
STD: Saturation-Transfer Difference
TEV: Tobacco Etch Virus
VS: Virtual screening
XP: Extra precision
