# Supplementary material for: In-silico guided chemical exploration of KDM4A fragments hits
Source: Clin Epigenetics. 2023 Dec 21;15:197. doi: 10.1186/s13148-023-01613-7 (PMC10740270; doi:10.1186/s13148-023-01613-7)
Supplement: Supplementary file 1 — Additional file 1. Supplementary Figures and Tables. [file 13148_2023_1613_MOESM1_ESM.docx]

**SUPPLEMENTARY INFORMATION**

**In-silico guided chemical exploration of KDM4A fragments hits**

Jessica Lombino^1,§^, Rosario Vallone^2,§^, Maura Cimino^3^, Maria Rita Gulotta^1^, Giada De Simone^1^, Maria Agnese Morando^2^, Raffaele Sabbatella^2^, Simona Di Martino^4^, Mario Fogazza^3^, Federica Sarno^5,6^, Claudia Coronnello^7^, Maria De Rosa^4^, Chiara Cipollina^3^, Lucia Altucci^5,8,9^, Ugo Perricone^1,*^, Caterina Alfano^2,*^

^1^Molecular Informatics Group, Fondazione Ri.MED, Palermo 90100, Italy. ^2^Structural Biology and Biophysics Unit, Fondazione Ri.MED, Palermo 90100, Italy. ^3^Target identification and screening Group, Fondazione Ri.MED, Palermo 90100, Italy. ^4^Medicinal Chemistry Group, Fondazione Ri.MED, Palermo 90100, Italy. ^5^Dipartimento di Medicina di Precisione, Università degli Studi della Campania "L. Vanvitelli" Napoli 80100, Italy. ^6^Current address: Department of Pathology and Medical Biology, University Medical Center Groningen, University of Groningen, 9713 GZ Groningen, The Netherlands. ^7^Advanced Data Analysis Group, Fondazione Ri.MED, Palermo 90100, Italy. ^8^BIOGEM, Ariano Irpino (AV) 83031, Italy. ^9^IEOS-CNR, Napoli 80100, Italy.

^§^These authors equally contributed to the work

*To whom correspondence should be addressed

[calfano@fondazionerimed.com](mailto:calfano@fondazionerimed.com); [uperricone@fondazionerimed.com](mailto:uperricone@fondazionerimed.com)

**A**

**B**

**Suppl. Fig. S1:** **Comparison of pharmacophore models generated from different PDB structures.** (A) ROC curves: five pharmacophore models, each generated from different PDB structure, were compared by evaluation of the ROC curve parameters. The pharmacophore model generated from 5VMP resulted the best in terms of AUC and EF. (B) Comparison of the chemotypes recognized by the two best performing pharmacophores 5VMP and 6H4U: the 6H4U-based pharmacophore model was also considerable in terms of ROC parameters, but it only recognized two chemotypes overlapping the pharmacophore map (while 5VMP was capable to identify 3 different chemotypes upon actives), and the phamacophore fit score was poor when compare to the 5VMP (46 vs 58) considering the same number of features (5). Another issue for 6H4U and the other 5 PDBs, also relies in the spatial dispersion of pharmacophore features, not focusing on the crucial interaction pattern known in the literature, and particularly important in the preliminary fragment screening approach chosen in this study.


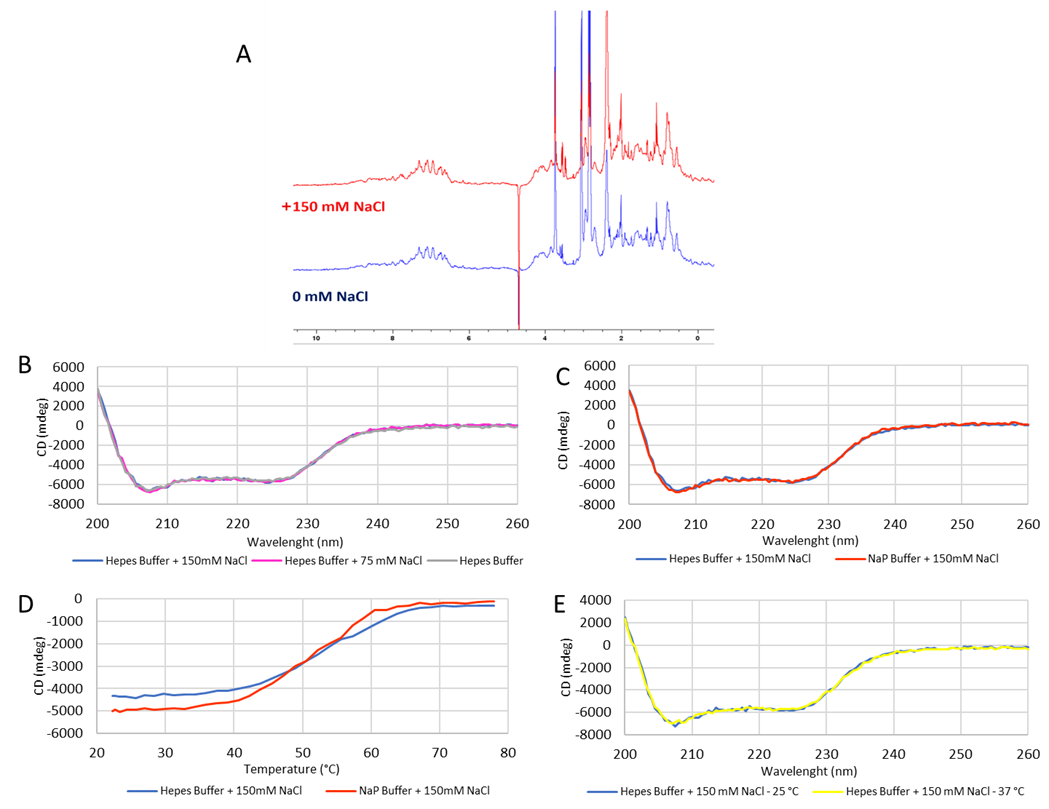


**Suppl. Fig. S2:** **Testing folding and stability of recombinant KDM4A.** Folding and stability of recombinant KDM4A, freshly prepared in-house to be used for both HTRF-based assay and BLI, was tested by far-UV CD spectroscopy and ^1^H NMR spectroscopy. ^1^H NMR spectra were characteristic of well folded proteins showing wide dispersion of the resonance signals. The CD far-UV spectra showed two strong negative bands with local minima at 208 and 222 nm indicating a high α-helical content, in agreement with the 3D X-ray structure of KDM4A. Thermal stability analysis was also conducted in the range 20–95°C by monitoring the dichroic signal at 222 nm, resulting in a melting temperature *T_m_* of about 55 °C. (A) 1D ^1^H NMR spectra at two different concentration of NaCl, 0 mM (in blue) and 150 mM (in red). (B) CD far-UV spectra in Hepes buffer at three different concentration of NaCl, 0 mM, 75 mM, and 150 mM. (C) CD far-UV spectra and (D) CD thermal denaturation curves in Hepes buffer and NaPh buffer, both supplemented with 150 mM NaCl. (E) CD far-UV spectra in Hepes buffer, supplemented with 150 mM NaCl, at both 25 and 37°C.


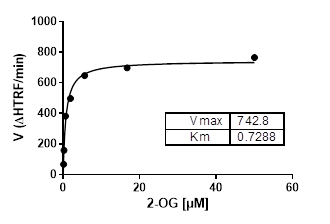


**Suppl. Fig. S3. KDM4A demethylase activity on H3K9me3 peptide in presence of 2-OG substrate** The enzymatic reaction was performed titrating 2-OG using concentrations ranging from 50 µM to 0.01 µM, and led to a K_m_ value of 0.73 ± 0.16 µM determined *via* Michaelis-Menten plot. Graph depicts mean of three replicates of the obtained percentage of inhibition at each tested concentration. Data were fitted using a Michaelis Menten plot. Calculation of K_m_ was performed by GraphPad Prism 9.0 software (GraphPad Software, Inc., San Diego, CA, United States).

The K_m_ was then used to set the 2-OG concentration in the HTRF-based screening. Indeed, the correlation between the enzyme K_m_ and the substrate concentration has a big impact on the type of inhibitors that can be identified during the screening.^1^ In our case, we then set 2-OG concentration at 0.5 µM.


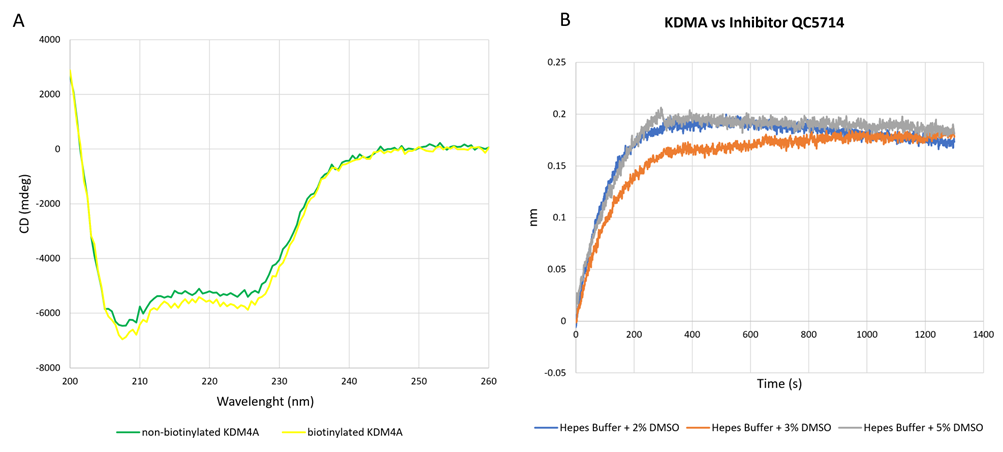


**Suppl. Fig. S4:** **Stability of biotinylated KDM4A for BLI experiments.** Since BLI experiments required biotin-labelled KDM4A, the secondary structure of biotinylated KDM4A was evaluated by far-UV CD spectroscopy. No significant change in the CD spectrum appeared compared to the non-biotinylated protein, confirming the applicability of the chosen technique to our purpose. The DMSO tolerability of KDM4A was tested by BLI using QC5714 as reference,^2^ given that all tested compounds in this study are dissolved in DMSO. The resulted BLI sensorgrams showed that KDM4A maintains almost the same binding capability for QC5714 at the 3 tested DMSO concentations, 2%, 3% and 5%. Accordingly, kinetic buffer supplemented with 5% DMSO was used to improve compounds solubility in all BLI experiments. (A) CD far-UV spectra of both free (green) and biotinylated (yellow) KDM4A. (B) BLI sensorgrams at different DMSO concentrations.


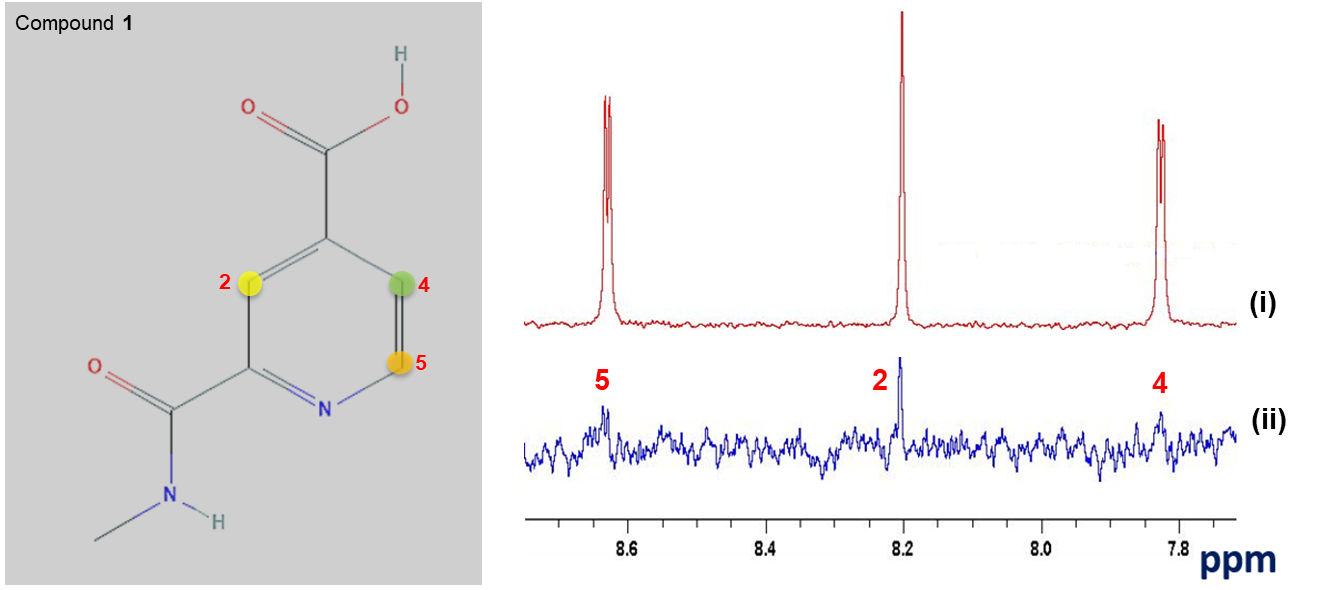


**Suppl. Fig. S5: STD-NMR analysis of compound 1.** STD-NMR experiment detected compound **1** binding to KDM4A. (i) The 1D NMR reference spectrum of compound **1**. (ii) The STD-NMR spectrum of compound **1** in the presence of KDM4A. The NMR spectrum was recorded at 1:50 receptor-to-ligand ratio. The protons in position 2,4,5 labelled with colors, exhibit an STD effect upon binding to KDM4A.

#

**Suppl. Table S1**. Centroids of clustered chemotypes from VS molecules. As appreciable from the variability of the chemotypes, the use of a pharmacophore approach before docking avoids bias in the virtual screening models towards a specific chemotype.

**Suppl. Table S2**. Per-residue interaction scores of compound **1**, analogues, and synthesized derivatives.

| Title | Structure | res:A501 Eint | res:A206 hbond | res:A206 Eint | res:A132 hbond | res:A132 Eint |
| --- | --- | --- | --- | --- | --- | --- |
| RIM571  Compound **1** |  | -103.25 | 0 | -48.19 | -0.50 | -6.82 |
| RIM675 |  | -26.923 | 0 | -2.486 | -1.000 | -7.622 |
| RIM235 |  | -30.491 | 0 | 3.021 | -0,46 | -4.959 |
| RIM702 |  | -15.772 | 0 | -5.275 | -0,747 | -4.747 |
| RIM233 |  | -25.598 | 0 | 6.673 | -0,417 | -4.973 |
| RIM234 |  | -28.773 | 0 | 4.259 | -0,461 | -4.851 |
| RIM673 |  | -25.835 | -0,268 | -8.667 | -0,875 | -2.059 |
| RIM515 |  | -28.403 | 0 | -0,788 | -1.000 | -6.660 |
| RIM559 |  | -3.676 | -0,5 | -12.977 | -1.094 | -3.332 |
| RIM703 |  | -14.366 | 0 | -4.423 | -1.000 | -6.476 |
| RIM511 |  | -2.721 | 0 | 0,677 | -1.000 | -3.693 |
| RIM709 |  | -28.131 | -0,43 | -9.584 | -0,988 | -2.376 |
| RIM680 |  | -27.227 | 0 | -7.353 | -1.260 | -5.621 |
| RIM684 |  | -26.501 | 0 | -2.936 | -1.591 | -5.196 |
| RIM683 |  | -27.628 | 0 | -3.021 | -1.084 | -5.291 |
| RIM704 |  | -23.079 | 0 | -4.403 | -0,83 | -3.691 |
| RIM479 |  | -64.296 | 0 | 0,527 | 0 | -0,085 |
| RIM278 |  | -27.155 | -0,183 | -3.040 | 0 | -1.119 |
| RIM229 |  | -146.832 | 0 | -23.691 | -0,496 | -4.969 |
| RIM699 |  | -112.384 | 0 | 24.463 | -1.000 | -7.835 |
| RIM653 |  | -184.697 | 0 | -74.326 | -0,465 | -6.590 |
| RIM694 |  | -114.972 | 0 | -25.230 | -0,967 | -7.396 |
| RIM692 |  | -138.031 | 0 | -23.350 | -1.000 | -7.630 |
| RIMJL13 (5a) |  | -12.303 | 0 | -5.814 | -1.000 | -6.479 |
| RIMJL16 (5b) |  | 25.424 | 0 | 1.053 | -0,75 | -5.398 |
| RIMJL14 (5c) |  | -27.296 | 0 | -0,512 | -1.000 | -4.227 |
| RIMJL21 (5d) |  | -22.931 | -0,383 | -10.956 | -0,57 | -1.458 |
| RIMJL17 (6a) |  | -103.480 | 0 | -49.226 | -0,479 | -6.655 |
| RIMJL18 (6b) |  | -128.853 | 0 | -21.078 | -1.000 | -6.479 |
| RIMJL20 (6c) |  | -122.671 | 0 | -20.702 | -1.000 | -7.118 |
| RIMJL25 (6d) |  | -103.259 | 0 | -48.682 | -0,5 | -7.264 |

**REFERENCES**

1. Yang, J., Copeland, R. A. & Lai, Z. Defining balanced conditions for inhibitor screening assays that target bisubstrate enzymes. *J. Biomol. Screen.* **14,** (2009).
2. Chen, Y. K. *et al.* Design of KDM4 Inhibitors with Antiproliferative Effects in Cancer Models. *ACS Med. Chem. Lett.* **8,** 869–874 (2017).
